# Reproducibility and rigor in rheumatology research

**DOI:** 10.3389/fmed.2022.1073551

**Published:** 2023-01-06

**Authors:** Fatima Alnaimat, Nadia J. Sweis, Jaleel Jerry G. Sweis, Christian Ascoli, Peter Korsten, Israel Rubinstein, Nadera J. Sweiss

**Affiliations:** ^1^Division of Rheumatology, Department of Medicine, The University of Jordan, Amman, Jordan; ^2^Department of Business Administration, King Talal School of Business Technology, Princess Sumaya University for Technology, Amman, Jordan; ^3^School of Medicine, The University of Jordan, Amman, Jordan; ^4^Division of Pulmonary, Critical Care, Sleep, and Allergy, Department of Medicine, University of Illinois Chicago, Chicago, IL, United States; ^5^Department of Nephrology and Rheumatology, University Medical Center Göttingen, Göttingen, Germany; ^6^Division of Rheumatology, Department of Medicine, University of Illinois Chicago, Chicago, IL, United States

**Keywords:** clinical trials, real-world data, registry, ethics, responsible conduct, research integrity

## Abstract

The pillars of scientific progress in rheumatology are experimentation and observation, followed by the publication of reliable and credible results. These data must then be independently verified, validated, and replicated. Peer and journal-specific technical and statistical reviews are paramount to improving rigor and reproducibility. In addition, research integrity, ethics, and responsible conduct training can help to reduce research misconduct and improve scientific evidence. As the number of published articles in rheumatology grows, the field has become critical for determining reproducibility. Prospective, longitudinal, randomized controlled clinical trials are the gold standard for evaluating clinical intervention efficacy and safety in this space. However, their applicability to larger, more representative patient populations with rheumatological disorders worldwide could be limited due to time, technical, and cost constraints involved with large-scale clinical trials. Accordingly, analysis of real-world, patient-centered clinical data retrieved from established healthcare inventories, such as electronic health records, medical billing reports, and disease registries, are increasingly used to report patient outcomes. Unfortunately, it is unknown whether this clinical research paradigm in rheumatology could be deployed in medically underserved regions.

## 1. Reproducibility and rigor in rheumatology research

In a manuscript presented by invitation at the 1992 Society of Exploration Geophysics (SEG) meeting (1), Claerbout and Karrenbach from Stanford University coined the term “reproducible research.” Claerbout, who pioneered the use of computers to process and filter seismic exploration data, required that Ph.D. dissertations of his students meet reproducibility standards, namely research data that could be independently replicated by others using a single computer command.

However, the terms reproducibility and replicability are not clearly distinguished in the literature. Barba (2) proposed a distinction between these terms. The term “reproducible research” was used when the data and computer codes were required to repeat the analysis and recreate the results. In contrast, the term “replicability” is used when a study reaches the same scientific conclusions as another study despite collecting new data and conducting new analyses.

Over half of researchers polled by Nature indicated that the scientific community was facing a severe reproducibility crisis (3). Richard Smith, a former BMJ editor, stated that it might be time to stop assuming that the research was indeed performed and accurately reported but instead to believe it was fraudulent until evidence to the contrary is presented (4). The pressure to publish new scientific discoveries in top journals significantly contributes to this crisis, particularly for early-career researchers attempting to establish a solid scientific record (5).

The number of publications in all research fields is rapidly increasing; rheumatology is no exception. For example, Cheng and Zhang (6) reported that there had been a threefold increase in the number of articles published in rheumatology over the past 20 years, making this specialty attractive for reproducibility evaluation.

After admitting to 3 years of data manipulation in a phase II study of using omalizumab to treat anti-citrullinated peptide antibody (ACPA)-positive rheumatoid arthritis, a senior rheumatology medicine investigator at a prestigious Dutch university hospital was dismissed (7). Senior academics could not replicate the research findings, and the publications that reported results from this trial were retracted.

Gasparyan et al. (8) searched PubMed, an electronic, publicly-accessible search engine on life sciences and biomedical topics, to assess the scope and magnitude of duplicate and retracted publications in rheumatology. Thirty-seven rheumatology journals ranked by the SCImago Journal Rank (SJR) indicator, a measure of the scientific influence of scholarly journals and listed in PubMed, were chosen. The total number of publications across all countries and the number of duplicate articles correlated significantly. It was found that the proportion of corrections published in 2013 accounted for 39% of all corrections, with 85% of these corrections coming from a single journal (8). Eighty percent of retracted articles were published between January 1, 2000, and December 31, 2013, coinciding with increased open-access publishing. The top three categories of retractions were comparative studies, randomized trials, and reviews, with articles from the United States being the most frequently duplicated and retracted.

## 2. Implementing reproducibility standards in rheumatology research

Randomized clinical trials (RCTs) are the gold standard for establishing clinical evidence in medicine. The strength and internal validity of the RCT stem from randomization’s ability to ascertain that no differences exist between the two treatment arms beside the administration of the treatment under consideration (9).

The significance of RCTs is contingent upon a transparent and precise results report. The published article must accurately reflect the study protocol, and the statistical plan must be adhered to or formally modified, with substantial justification for any deviations (10). Studies must report negative findings if encountered and not hide them.

The Consolidated Standards of Reporting Trials (CONSORT) (11), published for the first time in 1996, is one of the efforts to improve clinical trial reporting and transparency and provides evidence-based guidelines for reporting randomized trials. Hill et al. (12) compared 121 trials published between 1997 and 1998 to 119 studies published between 1987 and 1988 (before the CONSORT statement). They discovered an improvement in the quality of trial reporting. However, even in high-impact journals, methodological issues persisted (12).

## 3. Avoiding and identifying fraudulent research in rheumatology

The US National Institutes of Health (NIH) defines responsible conduct of research (RCR) as the practice of conducting scientific investigations with integrity. It applies established ethical and professional standards to all scientific research activities (13).

Responsible conduct and integrity training can reduce research misconduct and enhance scientific evidence (14). Conversely, positive and negative early research experiences can influence researchers’ adherence to ethical standards. Two-thirds of the participants in a survey study of researchers’ views on research integrity in Switzerland admitted they had no formal training in research integrity. They cited ambition and moral compass as the most significant determinants of the significance of ethical research (15). Therefore, in addition to securing research funding, it is necessary to investigate the actual research behavior and the factors influencing responsible research conduct.

Responsible conduct of research training is required for all US investigators funded by NIH and the National Science Foundation (NSF). However, after almost two decades of training, its efficacy in modifying research behavior remains inconsistent and limited (16).

## 4. Improving rigor of rheumatology research practices

Menke et al. (17) created an automated tool (SciScore) to rate how closely open-access scientific research articles adhere to rigor standards like those established by NIH and Research Resource Identifiers. In addition, the Rigor and Transparency Index (RTI), a yearly average score for journals based on their rigor and transparency, was introduced. Following the RTI’s introduction, studies more frequently meet the rigor criteria, but only about half of these criteria, such as blinding or power analysis, are consistently reported by authors. Interestingly, the RTI did not correlate with the Journal’s Impact Factor (17).

Disseminating published sources widely and contacting knowledgeable readers who can spot minor and major errors are the first steps in preventing misconduct. Research misconduct and the dissemination of false, inaccurate, or misleading information can occur in any publication. However, it has been noted that esteemed journals and periodicals using the open-access publishing model regularly retract or publish corrections (8). Additionally, the time between publication and retraction in journals with higher impact factors is shorter than in journals with lower impact, presumably because readers and authors pay less attention to the latter (18).

Due to the detrimental effects of “paper mills,” maintaining ethical standards and integrity in research practice is also challenging. These unethical organizations are adept at creating phony manuscripts that are then submitted to scholarly journals using plagiarism, fake results, and image falsification. According to one study, retractions of such fraudulent articles are rising (19).

The Rigor and Reproducibility policy of NIH, developed in 2016 in collaboration with the Nature Publishing Group and Science, exemplifies scholarly efforts to raise research integrity standards (20). These principles are based on the core set of standards for transparency in reporting detailed methods and rigorous statistical analyses, with an emphasis on data and material sharing, which states that all datasets on which the manuscript’s conclusions are based must be made available upon request, potentially through the deposition in publicly available repositories. Some journals in rheumatology, such as *Arthritis and Rheumatology*, require that authors make data and methods associated with the manuscript available to readers promptly without undue restrictions. Furthermore, the International Committee of Medical Journal Editors (ICMJE) formulated guidelines to help authors and editors produce understandable and reproducible medical journal articles (21).

## 5. Is it time to re-reproduce landmark studies?

Randomized controlled clinical trials are the gold standard for determining the efficacy of an intervention, but their applicability to real-world clinical settings can be limited (22). These trials are tedious, challenging, and expensive. In addition, the study’s inclusion criteria and patient selection can make it challenging to extrapolate the results to larger, more representative “real-world” patient populations.

Real-world data gathered from sources other than traditional clinical research settings, such as electronic health records (EHRs), billing information, and disease registries (22, 23) are increasingly used to supplement the outcomes of conventional clinical trials and provide an essential source for patient-reported outcome (PRO) measurements (24, 25). However, replicating the findings of clinical trials in real-world settings might not always be feasible. For example, a 2017 cross-sectional study (26) found that only 15% of 220 clinical trials published in journals with high-impact factors were replicable using insurance claims or EHRs.

Rheumatology research projects using real-world data have included several rheumatological diagnoses, but the majority of data focuses on rheumatoid arthritis (RA) (24). The data collected by RA registries in various countries (27–32) allows for a better understanding of the patient’s disease outcomes, responses to different therapies, particularly with the emerging use of biologics and biosimilars in rheumatology, and safety ofe these agents (33). A similar role of real-world data exists in other rheumatological diseases such as systemic lupus erythematosus, ankylosing spondylitis (AS), psoriatic arthritis (PsA), systemic sclerosis, idiopathic myositis, and vasculitis (24).

Real-world data was also used to develop treatment guidelines, such as the European Society for Clinical and Economic Aspects of Osteoporosis and Osteoarthritis (ESCEO) guidelines for knee osteoarthritis (34). These guidelines included agents such as chondroitin sulfate and glucosamine, which both lack RCT evidence for their use but have shown some efficacy in improving pain and function in real-life studies (34).

The Rheumatology Informatics System for Effectiveness (RISE) (35) is an extensive data registry created by the American College of Rheumatology (ACR) that includes over 1,000 rheumatology clinicians and 2.5 million patients. With the introduction of the RISE Pilot Project Award (36) by ACR’s RISE Registry and Rheumatology Research Foundation in 2022, early-career researchers and clinicians interested in conducting rheumatology research using real-world EHRs data now have access to the RISE registry.

Despite significant legal, logistical, and methodological challenges (25), the Nordic countries’ experience with collaboration across large population-based clinical rheumatology registries enabled the production of studies involving a large number of patients with inflammatory arthritis from different countries. Several projects are currently being performed based on this registry (37). Examples of large-scale observational studies from this project are a study of biologics used in 42,638 RA patients with a history of malignancy (38) and another study to assess the risk of neuroinflammatory events in 25,796 RA patients, 8,586 PsA patients, and 9,527 AS patients treated with TNF-alpha inhibitors (39).

Population-based observational studies provide insight into routine care delivery to a larger number of patients, including the elderly and those with comorbidities, and provide data on real-life long-term outcomes. Still, they have significant limitations. Certain difficulties are associated with using registry data (40). These studies have limited internal validity and can be potentially biased by treatment indications or practice changes (9).

Registries may not record longitudinal data, and data may not be readily accessible; therefore, they may not keep pace with clinical practice. Because registries are frequently limited to the specific indication they are intended to record, registry data may not accurately reflect the typical clinical application of a given drug or medical device. For instance, registry patients’ medication use is typically recorded as taken or not taken, but no comments can be made on a dose’s long-term effects. For example, registers’ patient-reported outcome measures may not capture all potential outcomes (40).

Investigators have less incentive in terms of return on investment. Therefore, sites spend less time with a patient registry, and researchers’ motivation often drives the registration process, particularly in academic institutions. Adequate compensation for data entry or an on-site researcher with prior competence may improve the rigor of data registration.

Missing data is common in patient registries and non-experimental studies that observe routine patient care (41). Imputing methods use model-predicted values to retain patients with missing data. Multiple imputations generate multiple data sets, each with a different imputed value for each missing variable, reflecting the uncertainty surrounding missing variable values. In contrast, in single imputation, the missing observation can be replaced with the sample mean or median, a predicted variable value (e.g., from a regression model), or even a study patient who matches the missing data on a set of chosen covariables. Multiple imputations outperform single imputations and are unlikely to introduce estimation bias because they use a robust model of missing data with good covariate (42).

Researchers must report potential biases from single imputations as well as technical limitations.

The European Medicines Agency (EMA) established the Patient Registry Initiative in 2015 to improve the utilization of patient registries and support the definition of study populations and study protocols with guidance on data gathering, data quality management, and data analysis (43).

## 6. Economic impact of irreproducibility in rheumatology research

The economic impact of reproducibility in biomedical research is enormous. Previous studies have shown that the prevalence of irreproducible preclinical research in the US alone exceeds 50%, with ∼28 billion US dollars spent yearly on preclinical research that is not reproducible (44). These low reproducibility rates erode new knowledge accumulation and contribute to appreciable delays and costs of drug development for patients in need. Thus, ignoring the lack of reproducibility in rheumatology research is both important and costly. Addressing this “reproducibility crisis” requires stakeholders to conduct unbiased, multi-faceted, root-cause analysis of irreproducible studies in this space that includes, but is not limited to, review of study design and data collection, analysis, and interpretation and proposing corrective measures to prevent recurrences.

## 7. Perspective and concluding remarks

The medical community is suffering from the COVID-19 pandemic crisis, and the rate of physician burnout has increased significantly. Many talented young investigators changed their career pathways and left academic medicine. There is a national and international shortage of physician-scientists in rheumatology and other specialties. The additional impact of the lack of rigor and reproducibility in rheumatology causes a threat to the future of academic rheumatology, medical education, and valid research which will ultimately impact the patient’s well-being and health. Providing outstanding scientific and evidence-based patient care remains our utmost goal in the field of rheumatology. A national effort supported by multiple stakeholders in academic medicine is urgently required. Standardizing the definitions of integrity, ethics, and rigor is a step forward in the right direction.

Implementing rigorous peer and journal-specific technical and statistical reviews of submitted manuscripts following reproducibility criteria, such as the Rigor and Reproducibility policy of the NIH can assist in improving rigor and reproducibility by removing major causes of irreproducibility, such as methodological flaws and inadequate or inaccurate reporting, and counteract the harmful effect of irreproducibility.

Research integrity, ethics, and responsible conduct training can reduce unethical research and improve scientific evidence. By adhering to the rigor guidelines and including a clear description in submitted works, the scientific community can more easily replicate or invalidate research findings. Real-world data makes it possible to replicate landmark studies and add patient outcomes in underrepresented populations. Whether this clinical research paradigm in rheumatology and other disciplines of medicine could be deployed in medically underserved regions of the world remains to be determined.

## Data availability statement

The original contributions presented in this study are included in the article/supplementary material, further inquiries can be directed to the corresponding author.

## Author contributions

FA, NS (2nd author), JS, and NS (7th author) searched the literature, drafted and revised the manuscript. CA, PK, and IR critically revised and edited the manuscript. All authors contributed to the article and approved the submitted version.
