# Corrigendum: Reproducibility and rigor in rheumatology research

**DOI:** 10.3389/fmed.2023.1267505

**Published:** 2023-08-15

**Authors:** Fatima Alnaimat, Nadia J. Sweis, Jaleel Jerry G. Sweis, Christian Ascoli, Peter Korsten, Israel Rubinstein, Nadera J. Sweiss

**Affiliations:** ^1^Division of Rheumatology, Department of Medicine, The University of Jordan, Amman, Jordan; ^2^Department of Business Administration, King Talal School of Business Technology, Princess Sumaya University for Technology, Amman, Jordan; ^3^School of Medicine, The University of Jordan, Amman, Jordan; ^4^Division of Pulmonary, Critical Care, Sleep, and Allergy, Department of Medicine, University of Illinois Chicago, Chicago, IL, United States; ^5^Department of Nephrology and Rheumatology, University Medical Center Göttingen, Göttingen, Germany; ^6^Division of Rheumatology, Department of Medicine, University of Illinois Chicago, Chicago, IL, United States

**Keywords:** clinical trials, real-world data, registry, ethics, responsible conduct, research integrity

In the published article, the last name for author “Israel Rubinstein” was incorrectly written as Rubenstein. The correct spelling is Rubinstein.

The authors apologize for this error and state that this does not change the scientific conclusions of the article in any way. The original article has been updated.

